# Effects of different feeding strategies on behavior and performance in broiler breeder pullets

**DOI:** 10.1016/j.psj.2024.104336

**Published:** 2024-09-17

**Authors:** R.A. van Emous, C. Kemp, J. van Meerveld, J. Lesuisse

**Affiliations:** ⁎ Department of Animal Nutrition, Wageningen Livestock Research, NL-6708 WD Wageningen, The Netherlands; † Aviagen Ltd, Newbridge, Edinburgh, Scotland, EH28 8SZ

**Keywords:** Broiler breeder, diet density, feeding frequency, behavior

## Abstract

This study examined the effects of different feeding strategies (diluted diets and feeding frequency) on the behavior and performance of broiler breeder pullets. A total of 3,200 1-day-old female pullets (Ross 308) were randomly distributed over 16 floor pens in 4 rooms and allocated to 1 of 4 treatments: (1) control diet once a day (**CON**), (2) 20% diluted diet once a day (20-ON), (3) 20% diluted diet twice a day (20-TW), and (4) 30% diluted diet twice a day (30-TW). All the pullets of the different treatments were fed to the same body weight (BW) profile. The 30-TW pullets had the highest and the CON pullets the lowest feed intake, with the 20-ON and 20-TW pullets showing intermediate values. Total water intake was the highest for the 30-TW pullets, followed by the 20-TW and 20-ON pullets, and was the lowest for the CON pullets (*P* < 0.001). The pullets fed twice a day had the highest total water-to-feed ratio, and the pullets fed once a day had the lowest ratio (*P* = 0.003). Feeding pullets twice a day yielded the highest average BW uniformity, while the pullets fed once a day had the lowest BW uniformity (*P* = 0.003). Total mortality was lower in the 20-TW and 30-TW pullets than in the 20-ON pullets, which was primarily caused by fewer dead and graded pullets. The pullets fed twice a day showed overall more eating, more drinking and less sitting, object pecking, and aggressive pecking. The behavior pattern during the daylight period was different for the pullets fed twice a day. Pullets on the diluted feeding strategies were less eager to approach the novel feeder and ate less feed (*P* = 0.002). In conclusion, feeding pullets with adjusted feeding strategies (feeding twice a day and up to 30% diluted diets) resulted in improved behavior and welfare expressed in decreased stereotypic pecking behavior, and lower eagerness to approach the novel feeder with lower feed intake, with improved BW uniformity and decreased mortality.

## INTRODUCTION

Decades of intensive genetic selections have given broilers the potential for highly efficient growth, and fast-growing Ross 308 broilers can reach a body weight (BW) of 2.3 kg in 35 d (Aviagen, 2022). The parent stock of broilers (i.e., broiler breeders) produce these efficient broilers, and they share the same genetic potential for growth and feed efficiency (Havenstein et al., 2003a,b; Renema et al., 2007; Zuidhof et al., 2014). Feeding pullets and breeders ad libitum makes breeders overweight, resulting in severe negative effects on health and reproduction (Katanbaf et al., 1989; Savory et al., 1993; Heck et al., 2004). These negative effects can be prevented by feeding breeders a controlled amount of feed (van Emous, 2022). This feed control, however, involves various welfare issues, such as stereotypic pecking behavior toward the equipment of the house, empty feeders, empty drinkers, and tail licking/sucking (Hocking et al., 1996, 2001; Savory and Kostal, 1996; de Jong et al., 2002; Nielsen et al., 2011; van Emous et al., 2015). In the literature, it is repeatedly mentioned that the feed control level in breeder pullets is between 67% and 75% of the feed intake during the rearing period (Savory et al., 1993; Savory and Kostal, 1996; de Jong et al., 2002). Recently, Carney et al. (2022) conducted a comparison of 4 distinct broiler breeder strains (1957, 1978, 1995, and 2015), with diets provided either ad libitum or with restrictions. To maintain the target body weight in the restricted-fed pullets across the various strains, feed restriction levels were set at 1%, 43%, 70%, and 75% for the 1957, 1978, 1995, and 2015 strains, respectively. This estimation is, however, based on a comparison of ad libitum- and restricted-fed birds of the same age but with different BW, leading to an overestimation of the feed restriction level. An improved method was introduced by Arrazola (2018), who estimated the feed restriction levels by comparing the target feed intake of modern broiler breeders and modern broilers of the same BW. Based on this method, van Emous and Mens (2022) developed a more realistic estimation of feed restriction by implementing field-confirmed energy content of breeder and broiler diets, resulting in a 47% energy restriction level in the rearing phase.

One of the methods for reducing the effects of feed control on unwanted behavior is increasing the daily feed volume by diluting diets with, for example, wheat bran, and oat hulls (e.g., de Jong et al., 2005a; Tahamtani et al., 2020, Mens et al., 2022). Diet dilution with fibers reduced object pecking during the first part of the rearing phase (de Jong et al., 2005a), reduced spot pecking and heterophil-to-lymphocyte ratio (Hocking et al., 2004), increased dustbathing behavior, and reduced stereotypic pecking (Nielsen et al., 2011). To the authors’ knowledge, the use of wheat straw pellets as a dilution ingredient in pullet diets has not yet been studied. Wheat straw is an insoluble fiber source of uniform quality and has a high water-holding capacity. It has been suggested that the accumulation of insoluble fiber in the gizzard triggers a temporary satiety due to the regulatory role of the gizzard in feed passage (Hetland et al., 2004). Nielsen et al. (2011) tested diets with high levels of soluble (sugar beet and potato pulp) or insoluble (oat hulls) fiber during the rearing phase and did not observe stereotypic pecking, while this was frequently observed in pullets fed the control diets. The pullets fed the insoluble fiber diets also showed more dust bathing, comfort behavior, and foraging, which were interpreted as positive welfare indicators.

Under commercial circumstances, pullets are fed 1 meal a day (Europe) or follow feed programs with feed-less days, such as a skip-a-day program or a program with 5 feed days and 2 feed-less days (e.g., the United States and Asia). In contrast, it can be argued that feeding twice a day would correspond better to the animals’ natural feeding behavior and activity pattern. In a study by Collias and Collias (1967), the ancestors of modern breeders (red junglefowls) were observed in their natural habitat in North-Central India, and they found that these birds were active during dawn (eating, drinking, and egg laying) and dusk (eating, drinking, and mating) and inactive around noon. De Jong et al. (2005b) investigated the effects of feeding twice versus once a day, in addition to scattering feed in the litter (spin feeder) or feeder pans, on stress and hunger during the rearing period. They found decreased object pecking in pullets fed twice a day in the litter as compared with pullets fed once a day, which is an indication of improved welfare. More recently, in a study by Mens et al. (2022), feeding pullets twice a day had only minor effects on the welfare of breeder pullets. However, they found adjusted behavioral patterns during the day.

The combination of feeding twice a day and diets diluted with wheat straw pellets (up to 30%) has not been studied before. Based on earlier pilot studies under commercial circumstances, it was expected that the 30% diluted diets could result in semi-unrestricted access to feed during the light period. Therefore, the objective of this study was to evaluate the effects of different feeding strategies (diluted diets and feeding frequency) on the behavior and performance of broiler breeder pullets.

## MATERIALS AND METHODS

### Experimental Design

The experimental design was a randomized block design with 4 treatments: control diet once a day (CON), 20% diluted diet once a day (20-ON), 20% diluted diet twice a day (20-TW), and 30% diluted diet twice a day (30-TW), with 4 replicates per treatment. Pens were allocated to 4 rooms, and the different treatments were placed in the same rooms to avoid confounding effects. Any interactions between pens were minimized by a 30-60 cm high metal sheet at the bottom of the mesh sidewalls of the pens. The diets were formulated according to the Aviagen nutritional specifications using the European 5-stage rearing program (Aviagen, 2021). All the diets were formulated according to the WPSA energy and Amino Dat 5.0 amino acid data, and the standard Aviagen-EPI premix was used. Wheat straw pellets (3.0% CP, 37.7% CF, 68.8% NDF, 44.5% ADF, 6% ADL, 0.5% Calcium, 1.1% Potassium, without energy or dig amino acid contribution, 6 mm pellets) were used in the feed formulation to achieve the nutrient dilution (Table 1, Table 2). Nutrient constraints were kept at the same ratio to energy as in the control diet and reduced in line with the nutrient dilution. Premix, calcium, available phosphorus, and digestible threonine were diluted 10% less than the nutrient dilution to create a safety margin. Sodium and chloride levels in the diluted diets were not reduced to support water intake and prevent constipation. The experimental diets were produced by ForFarmers, Lochem, The Netherlands by mixing all raw materials prior to the roller mill and producing a crumble (Starter 1 diets) or coarse mash (all other diets). Particle size distribution was very similar for all treatments. Feeding twice a day started on d 8, and the pullets fed twice a day (20-TW and 30-TW) were fed at 0,700 h (50% of the daily feed allocation) and 1,100 h (50% of the daily feed allocation). All pullets received the same control starter-1 diet ad libitum during the first 7 d. To avoid major differences in the amount of feed and to prevent potential constipation caused by switching to the final dilution level, the dilution level was gradually increased for the 20-ON, 20-TW, and 30-TW pullets. These pullets received a starter-1 diet with 10% dilution from d 15 to d 21. From d 22 to d 41, the CON pullets received a control starter-2 diet. The 20-ON and 20-TW pullets received a starter-2 diet with 20% dilution from d 22 to d 41. The 30-TW pullets received a starter-2 diet with 20% dilution from d 22 to d 28 and a starter-2 diet with 30% dilution from d 29 to d 41. The pullets were fed according to the following feeding program: starter-1 (up to 3 wk of age [WOA]), starter-2 (3–6 WOA), grower (6–15 WOA), and developer (15–20 WOA). To support gut health and BW uniformity during the transition to the next feeding phase, the feed was mixed in 4 d to the next phase; that is, d 1: 100% starter-2, d 2: 67% starter-2 and 33% grower, d 3: 33% starter-2 and 67% grower, and d 4: 100% grower. To avoid major transitions in feed quantity, the dilution level of the prebreeder diets was gradually decreased for the pullets fed the diluted diets during the rearing phase (Table 2). The 20-ON and 20-TW pullets received a 10% diluted prebreeder diet for 2 wk, and the 30-TW pullets received a 20% diluted prebreeder diet followed by a 10% diluted prebreeder diet for 1 wk. From 20 WOA, all breeders received 1 meal a day, and from 24 WOA all breeders were fed the same breeder-1 and breeder-2 diets. From 26 WOA, males were fed a male diet (2,650 kcal/kg AMEn; 12.3% CP; 0.33% dig. Lys; 0.55% dig. M+C; 0.69% Ca; 0.34% aP).

**Table 1:** Overview of the diets fed in the different treatments, dietary ingredients, and calculated nutrients of the rearing phase diets (%).

	Starter-1		Starter-2			Grower			Developer		
Item	CON	10% DIL	CON	20% DIL	30% DIL	CON	20% DIL	30% DIL	CON	20% DIL	30% DIL
Age fed (d)											
CON^1^	0–21	-	22–41	-	-	42–105	-	-	106–140	-	-
20-ON^1^	0–14	15–21	-	22–41	-	-	42–105	-	-	106–140	-
20-TW^1^	0–14	15–21	-	22–41	-	-	42–105	-	-	106–140	-
30-TW^1^	0–14	15–21	-	22–28	29–41	-	-	42–105	-	-	106–140
Ingredient											
Wheat	29.4	24.4	32.8	26.8	22.7	31.2	24.6	21.6	33.0	25.4	22.1
Corn	29.0	24.0	34.0	26.0	23.0	32.0	25.0	21.0	33.0	26.0	22.0
Soybean meal 46%	-	-	-	-	-	2.2	1.5	1.0	1.4	0.8	1.1
Soybean meal 48%	24.8	19.3	15.6	12.7	10.8	-	-	-	-	-	-
Rapeseed meal	2.0	2.0	3.0	3.0	3.0	5.0	3.0	2.0	4.0	2.0	1.0
Sunflower meal 31%	-	-	-	-	-	15.0	13.2	13.2	15.0	13.5	12.0
Sunflower meal 36%	4.5	7.8	8.1	5.6	5.0	-	-	-	-	-	
Wheat bran 9% CF	-	-	-	-	-	5.0	5.0	5.0	5.0	5.0	5.0
Oat hulls	1.5	2.5	-	-	-	3.3	1.9	1.0	1.9	1.0	1.0
Wheat straw pellets	1.0	11.0	1.0	21.0	31.0	1.0	21.0	31.0	1.0	21.0	31.0
Corn oil	2.5	3.6	1.0	1.0	1.0	1.0	1.0	1.0	1.5	1.5	1.5
Monocalcium phosphate	1.80	1.82	1.69	1.56	1.39	1.61	1.48	1.31	1.59	1.47	1.30
Limestone	1.56	1.42	1.35	0.96	0.67	1.22	0.86	0.60	1.13	0.78	0.54
Sodium chloride	0.27	0.24	0.26	0.21	0.18	0.25	0.19	0.17	0.25	0.20	0.17
Sodium carbonate	0.15	0.18	0.16	0.21	0.23	0.15	0.21	0.23	0.16	0.21	0.23
Premix^2^	0.30	0.30	0.30	0.27	0.24	0.30	0.27	0.24	0.30	0.27	0.24
Lysine sulphate	0.27	0.31	0.05	0.04	0.04	0.08	0.08	0.08	0.12	0.11	0.09
DL-Methionine	0.30	0.27	0.15	0.13	0.11	0.12	0.10	0.08	0.11	0.09	0.09
L-Threonine	0.11	0.18	0.08	0.12	0.12	0.08	0.12	0.11	0.09	0.12	0.11
Valine	0.01	0.02	-	-	-	-	-	-	-	-	-
Choline chloride	0.07	0.07	0.07	0.07	0.07	0.07	0.07	0.07	0.07	0.07	0.07
Mannan oligosaccharide	0.10	0.10	-	-	-	-	-	-	-	-	-
Organic acids	0.40	0.40	0.40	0.40	0.40	0.40	0.40	0.40	0.40	0.40	0.40
Calculated content^3^											
AME_n_ WPSA (kcal/kg)	2,810	2,520	2,800	2,240	1,960	2,600	2,080	1,820	2,700	2,160	1,890
Crude protein	19.9	18.0	17.7	14.8	13.3	14.0	11.8	10.8	14.0	11.8	10.7
Crude fat	4.5	5.4	3.2	3.0	2.9	3.4	3.1	3.0	3.8	3.6	3.5
Crude fiber	3.9	8.1	4.1	10.8	14.3	7.3	13.4	16.6	6.6	13.0	16.1
Crude ash	6.8	7.2	6.3	6.5	6.5	6.1	6.3	6.3	5.9	6.2	6.2
Calcium	1.05	1.05	0.95	0.85	0.76	0.90	0.81	0.72	0.90	0.81	0.72
Total phosphorus	0.78	0.78	0.76	0.67	0.60	0.75	0.66	0.60	0.77	0.68	0.61
Available phosphorus	0.50	0.50	0.47	0.42	0.38	0.45	0.41	0.36	0.45	0.41	0.36
Sodium	0.20	0.20	0.20	0.20	0.20	0.20	0.20	0.20	0.20	0.20	0.20
Chloride	0.23	0.23	0.23	0.23	0.23	0.23	0.23	0.23	0.23	0.23	0.23
Potassium	0.89	0.90	0.77	0.84	0.87	0.63	0.73	0.78	0.66	0.76	0.81
dEB (mEq/kg)	250	252	220	237	245	183	208	222	190	216	229
Linoleic acid	2.35	2.74	1.70	1.44	1.32	1.75	1.50	1.38	2.02	1.77	1.64
ADL	1.1	1.9	1.3	2.2	2.7	2.1	2.9	3.3	2.0	2.8	3.2
NDF	12.1	18.8	12.5	23.7	29.5	17.3	27.7	32.9	16.5	27.1	32.5
ADF	5.4	10.2	5.6	13.4	17.4	8.7	16.0	19.7	8.2	15.6	19.3
Dig. amino acids (SID)											
Dig. Lys	1.00	0.90	0.72	0.58	0.50	0.48	0.38	0.34	0.48	0.38	0.34
Dig. Met + Cys	0.84	0.76	0.68	0.54	0.48	0.58	0.46	0.41	0.58	0.46	0.41
Dig. Thr	0.70	0.70	0.60	0.54	0.48	0.48	0.43	0.38	0.48	0.43	0.38
Dig. Val	0.81	0.73	0.72	0.57	0.50	0.56	0.45	0.40	0.56	0.45	0.39
Dig. Trp	0.22	0.19	0.19	0.15	0.13	0.14	0.12	0.10	0.14	0.11	0.10
Dig. Arg	1.17	1.05	1.02	0.81	0.71	0.78	0.62	0.56	0.76	0.62	0.55
Dig. Leu	1.34	1.16	1.20	0.95	0.831	0.91	0.72	0.63	0.91	0.72	0.63
Dig. Isoleu	0.72	0.63	0.62	0.50	0.43	0.46	0.36	0.32	0.45	0.36	0.32
Particle size distribution	Crumble	Crumble	Mash	Mash	Mash	Mash	Mash	Mash	Mash	Mash	mash
>3.15 mm	14.0	29.2	13.3	10.8	11.1	9.3	9.6	9.8	7.6	6.9	6.1
1–3.15 mm	51.8	57.1	63.0	58.8	57.0	64.6	59.1	58.3	65.0	62.9	59.8
<1.0 mm	26.4	13.9	23.7	30.4	31.9	26.1	31.3	31.9	27.4	30.3	34.2

1 CON: control diets; 20-ON: 20% diluted diets once a day; 20-TW: 20% diluted diets twice a day; 30-TW: 30% diluted diets twice a day.

2 Provided per kilogram of a complete diet: vitamin A, 10,000 IU; vitamin B1, 7.0 mg; vitamin B2, 20.0 mg; vitamin B3, 29.3 mg; vitamin B5, 90.0 mg; vitamin B6, 10.0 mg; vitamin B9/B11, 5.0 mg; vitamin B12, 0.08 mg; vitamin D3, 2,000 IU; vitamin 25-hydroxycholecalciferol, 0.03 mg; vitamin E, 120.0 mg; vitamin H, 0.6 mg; vitamin K3, 12.0 mg; iron, 50.0 mg; copper, 15.0 mg; manganese, 120.0 mg; zinc, 90.0 mg; iodine, 2.0 mg; selenium, 0.4 mg.

3 WPSA and Amino Dat matrix values were used for diet formulation.

**Table 2:** Overview of the diets fed in the different treatments, dietary ingredients, and calculated nutrients of the laying phase diets (%).

	Prebreeder			Breeder-1	Breeder-2
Item	CON	10% DIL	20%DIL		
Age fed (d)					
CON^1^	141–168	-	-	169–224	225–280
20-ON^1^	-	141–154	-	155–224	225–280
20-TW^1^	-	141–154	-	155–224	225–280
30-TW^1^	-	148–154	141–147	148–224	225–280
Ingredient					
Wheat	35.8	31.9	28.2	25.4	27.0
Corn	35.0	32.0	28.0	40.0	40.0
Soybean meal 48%	6.8	6.0	5.4	15.4	12.3
Sunflower meal 31%	10.2	3.0	4.1	7.4	8.2
Sunflower meal 36%	4.1	9.1	6.8	-	-
Wheat straw pellets	1.0	11.0	21.0	-	-
Corn oil	1.7	1.5	1.5	2.4	-
Sunflower oil	-	-	-	-	2.5
Monocalcium phosphate	1.20	1.23	1.11	1.22	1.13
Limestone	0.50	0.50	0.50	-	-
Limestone coarse	2.44	2.37	1.91	6.88	7.45
Sodium chloride	0.25	0.23	0.20	0.23	0.22
Sodium carbonate	0.16	0.18	0.21	0.14	0.14
Premix^2^	0.30	0.30	0.30	0.30	0.30
Lysine sulphate	-	-	-	0.00	0.01
DL-Methionine	0.12	0.11	0.10	0.16	0.13
L-Threonine	0.07	0.12	0.11	0.08	0.10
Choline chloride	0.08	0.08	0.08	0.12	0.12
Organic acids	0.40	0.40	0.40	0.40	0.40
Calculated content^3^					
AMEn WPSA (kcal/kg)	2,800	2,520	2,420	2,800	2,800
Crude protein	14.4	13.3	12.2	15.6	14.6
Crude fat	3.9	3.5	3.4	4.7	4.7
Crude fiber	5.0	7.9	11.3	3.5	3.5
Crude ash	7.0	7.4	7.3	10.8	11.2
Calcium	1.50	1.50	1.35	3.00	3.20
Total phosphorus	0.64	0.61	0.56	0.61	0.59
Available phosphorus	0.35	0.35	0.32	0.36	0.34
Sodium	0.20	0.20	0.20	0.18	0.18
Chloride	0.23	0.23	0.23	0.21	0.21
Potassium	0.67	0.70	0.75	0.71	0.66
dEB (mEq/kg)	193	202	214	201	189
Linoleic acid	2.07	1.85	1.72	2.49	2.84
ADL	1.4	1.8	2.3	0.9	0.9
NDF	13.1	18.4	24.1	11.0	10.6
ADF	6.3	9.8	13.8	4.7	4.7
Dig. Lysine	0.48	0.43	0.38	0.62	0.56
Dig. Met + Cys	0.58	0.52	0.46	0.62	0.57
Dig. Thr	0.49	0.49	0.44	0.55	0.53
Dig. Val	0.59	0.53	0.47	0.64	0.60
Dig. Trp	0.15	0.14	0.12	0.16	0.15
Dig. Arg	0.83	0.75	0.66	0.90	0.84
Dig. Leu	0.98	0.88	0.78	1.11	1.04
Dig. Isoleu	0.50	0.45	0.40	0.56	0.52
Particle size distribution	mash	mash	mash	mash	mash
>3.15 mm	11.9	13.3	8.8	8.5	8.0
1.0–3.15 mm	64.9	65.4	65.8	70.3	75.4
<1.0 mm	23.2	21.3	25.4	21.3	16.6

1 CON = control diets; 20-ON = 20% diluted diets once a day; 20-TW = 20% diluted diets twice a day; 30-TW = 30% diluted diets twice a day.

2 Provided per kilogram of a complete diet: vitamin A, 10,000 IU; vitamin B1, 7.0 mg; vitamin B2, 20.0 mg; vitamin B3, 29.3 mg; vitamin B5, 90.0 mg; vitamin B6, 10.0 mg; vitamin B9/B11, 5.0 mg; vitamin B12, 0.08 mg; vitamin D3, 2,000 IU; vitamin 25-hydroxycholecalciferol, 0.03 mg; vitamin E, 120.0 mg; vitamin H, 0.6 mg; vitamin K3, 12.0 mg; iron, 50.0 mg; copper, 15.0 mg; manganese, 120.0 mg; zinc, 90.0 mg; iodine, 2.0 mg; selenium, 0.4 mg.

3 WPSA and Amino Dat matrix values were used for diet formulation.

### Housing and Management

A total of 3,200 Ross 308 broiler breeder pullets were housed at Spelderholt, the Aviagen trials facility in Lelystad, the Netherlands. The day-old chicks DOCs (200/pen) were allotted to 16 floor pens (3.05 × 7.00 m: 21.35 m^2^) in 4 identical climate-controlled rooms. At 20 WOA, the number of breeders was reduced randomly to 155. Males were reared separately in a compartment in the same pullet house, and 15 males per pen were placed at 20 WOA. The pens contained an elevated floor (3.05 × 1.20 m) with plastic slats, and wood shavings were used as litter on the remaining area (1.5 kg/m^2^). During the first 21 d, crumble feed was daily provided manually on 3 red plates per pen and chick paper (first 5 d) during the light period. From d 22 onward, coarse mash feed was daily distributed manually in a chain feeder (length 10.2 m) during the light period. From week 5 onward, feed was daily provided automatically by the chain feeder during the dark period. Feed allocation was measured automatically by a weekly calibrated system. The first feeding time (for all pullets) was at 0,700 h, and the second feeding time (for the 20-TW and 30-TW pullets) was at 1,100 h. Water was supplied ad libitum (during the light period) by a drinking system with 22 drink cups above the slatted floor and a drinking system with 22 drink cups above the litter. The latter system was removed at 5 WOA when the pullets were used to the drinking system on the slats. The breeder pullets remained in the same pens throughout the entire experiment and therefore had the same feed and water system during the production phase.

During the experiment, all birds of the different treatments were maintained on the same target body weight (BW). Feed allocation was weekly adjusted to the predetermined body growth curve (Aviagen, 2021). The pullets had unlimited access to pecking blocks (Pickblock Medium, Crystalyx Products GMBH, Münster, Germany) as distraction material during the entire rearing phase. Room temperature was maintained at 33.5°C during the first 2 d, and from d 3 onward, the temperature was gradually reduced to 20°C at wk 4. The pullets were reared following a photoperiod of 23L:1D (60 lx; 2,700 K) for the first 3 d, which was gradually reduced to a photoperiod of 8L:16D (5 lx; 4,500 K) at wk 3. Breeders were photostimulated with 11 h of light at 21 WOA (10 lx; 2,7,000 K), and the day length was gradually increased by 1 h per week to 13L:11D at week 23 (30 lx; 3,400 K). This photoperiod was maintained until the end of the study at 40 WOA. Outside each pen, adjacent to the slats, nest boxes were available from 22 WOA. Pullets were nonbeak trimmed and vaccinated according to a standard commercial protocol (Veterinary Center Someren, The Netherlands).

The study was approved by the welfare and compliance department of Aviagen and was conducted under welfare statement V51801 of Aviagen and audited by the IKB and NVWA.

### Observations

*Feed allocation.* Feed allocation was precalculated based on the daily energy intake of earlier diet studies at the same location. If needed, feed allocation was adjusted to maintain BW profiles based on weekly body measurements. Total feed allocation was calculated based on the daily feed allocation.

*Nutrient intake.* The intake of total energy (AMEn), crude protein, digestible lysine, and digestible methionine + cystine was calculated using the feed allocation per phase (starter-1, starter-2, grower, and developer) multiplied by the nutrient content.

*Water intake and water-to-feed ratio.* The water intake was automatically measured with a flow meter daily per pen and calculated per week, phase, and rearing period. The water-to-feed ratio was calculated by dividing the total water intake by the total feed intake per week, phase, and rearing period.

*Total daily water and feed intake.* The total daily water and feed intake per day was calculated by summing the feed and water intake.

*BW and BW uniformity.* To monitor BW and BW gain, a minimum of 50 females per pen were individually weighed weekly 5 h after feeding. Flock uniformity (BW CV%) was determined by calculating the standard deviation (SD) of BW divided by the average BW.

*Mortality.* Mortality and cause of mortality were recorded daily per pen to calculate total mortality and cause of mortality. Mortality was categorized into leg problems, culling (weak or sick), grading (too heavy or too light), pecking, and dead in pen (e.g., Sudden Death Syndrome, heart failure).

*Behavior.* Home pen behavior of the pullets was observed by live scan sampling of each pen at 6, 10, 14, and 18 WOA. At each age, observations were carried out during 1 d by 2 trained observers, both of whom observed all pens per session. Observers switched rooms between observation sessions. On each day, the observations were distributed across 4 sessions throughout the light period (starting at 0,730, 0,930, 1,130, and 1,330 h) with lights on between 0,700 and 1,500 h (8 h of light). Each session started with 2 min of habituation time per compartment, during which time the observer walked slowly between the pens. Feeding, drinking, foraging, standing, walking, sitting, comfort behavior (preening, nibbling, stroking, wing flapping, and stretching), dustbathing, feather pecking, object pecking, block pecking, and aggressive pecking were scored by counting the number of birds performing these different behaviors according to the ethogram previously described by van Emous et al. (2015). Feeding was recorded only when feed was available. During feed availability, object pecking was defined as pecking at the pen or equipment, and when the feed chains were empty, pecking at the feeder was also scored as object pecking.

*Novel food test.* The test was executed at 12 and 17 WOA. Feeders and feed were initially novel to the pullets at 12 WOA but not at 17 WOA, when the same feeders and feed were used. The test was performed at 2 time points per day (between 1,000 and 1,200 h and between 1,400 and 1,600 h) 3 h after the feeding moments. During each test, 2 pens per treatment, 1 per room, were tested, resulting in 8 pens per time point. All pens were tested once per day, and the order of pens was randomized and predetermined in advance. The pullets were presented with 2 feeders per pen (yellow round feeder, 30 cm in diameter and 6.5 cm in height) filled with 500 g feed (pellet feed). The pullets were allowed to eat for 2 min, after which the feeders were removed from the pen. The latency of the first pullet to approach the feeder and the number of pullets at the feeder after 1 and 2 min were recorded. Leftover feed from both feeders in each pen was weighed to measure the feed intake. All observations were performed live and by 2 trained observers.

*Egg Production Traits.* Eggs were collected, graded, and recorded daily per pen. The total number of settable (above 50 g), small (under 50 g), double-yolk, abnormal-shell, dirty, and floor eggs were calculated per pen and per week for the entire production period. All hatching eggs (settable) were weighed 5 d a week. The average egg weight was calculated based on the entire laying period.

### Statistical Analysis

The data were analyzed using Genstat statistical software (Genstat, 2022). A statistically significant difference was declared for *P* < 0.05, with 0.05 ≤ *P* < 0.10 considered a tendency. Response variables with regard to total feed and nutrient intake, mortality, cause of mortality, and egg performance traits were analyzed using the ANOVA (Analysis of Variance) procedure of Genstat according to the following model:

$$Y_{\text{ijk}}=\mu +R_{i}+F_{j}+\varepsilon_{\text{ijk}}$$

where Y_ijk_ is the response variable, μ is the overall mean, R_i_ is the random effect of the room (i = 1–4), F_j_ is the effect of the feeding program (CON, 20-ON, 20-TW, 30-TW; j = 1–4), and Ɛ_ijkm_ is the residual error term. Behavior traits were analyzed using the GLMM (Generalized Linear Mixed Model) procedure using logistic regression and a Poisson distribution of Genstat. The room was included in the model as a random term. The statistical model for the water intake, water-to-feed ratio, BW, BW uniformity, behavior traits, feed in the chain, and novel food test included the age or phase as a fixed effect. The pen was the experimental unit, and parameters were tested for normality before the analysis.

## RESULTS

### Feed Allocation and Nutrient Intake

**Figure 1:**
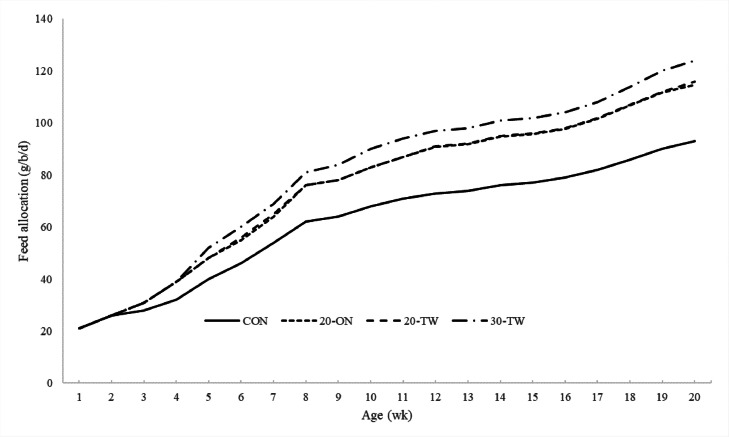
Effect of feeding strategies on feed allocation (g/b/d + SEM).

Feed allocation was the highest for the 30-TW pullets, followed by the 20-ON and 20-TW pullets, and was the lowest for the CON pullets (*P* < 0.001) (Table 3; Figure 1). This represents, respectively, a 30%, 22%, and 22% increase compared with the CON pullets. The intake of energy, digestible lysine, and digestible methionine + cystine intake was the lowest for the 30-TW, followed by the 20-ON and 20-TW pullets, and was the highest for the CON pullets (*P* < 0.001).

**Table 3:** The effects of different feeding strategies on total feed intake, energy intake, CP intake, dig. Lysine intake, and dig. Met + Cys intake from 0 to 20 WOA.

Treatment^1^	Feed intake (kg/b)	AMEn intake (kcal/b)	CP intake (g/b)	Dig. Lys intake (g/b)	Dig. M+C intake (g/b)
CON	8.69^c^	23,181^a^	1278.4^c^	46.44^a^	52.62^a^
20-ON	10.60^b^	22,833^b^	1319.4^a^	45.74^b^	51.84^b^
20-TW	10.63^b^	22,912^b^	1324.0^a^	45.90^b^	52.02^b^
30-TW	11.31^a^	21,556^c^	1295.7^b^	43.37^c^	49.02^c^
SEM	0.024	50.2	2.79	0.091	0.110
*P*-value	<0.001	<0.001	<0.001	<0.001	<0.001

a-c Means within a column with no common superscript differ (*P ≤* 0.05).

1 CON: control diet; 20-ON: 20% diluted diet fed once a day; 20-TW: 20% diluted diet fed twice a day; 30-TW: 30% diluted diet fed twice a day.

### Water Intake and Water-to-Feed Ratio

A tendency to a higher average water intake was found in the starter-1 phase for the 20-ON and 20-TW pullets compared with the CON pullets (*P* = 0.073; Table 4). The 30-TW pullets did not differ from the pullets receiving the other treatments. The average water intake in the starter-2 phase was 86.7, 92.3, 98.1, and 103.8 ml/b/d for the CON, 20-ON, 20-TW, and 30-TW pullets, respectively, showing a difference between all treatments (*P* < 0.001). The average water intake in the grower phase was 104.5, 135.8, 143.7, and 160.2 ml/b/d for the CON, 20-ON, 20-TW, and 30-TW pullets, respectively, showing a difference between all treatments (*P* < 0.001). In the developer phase, the CON pullets had the lowest water intake, which differed from that in the other treatments (*P* < 0.001). The 30-TW pullets had the highest water intake, and the water intake of the 20-ON and 20-TW pullets was between that of the CON and 30-TW pullets. The average water intake in the entire rearing phase was 106.4, 128.8, 135.7, and 148.4 ml/b/d for the CON, 20-ON, 20-TW, and 30-TW pullets, respectively, showing a difference between all treatments (*P* < 0.001). The average water-to-feed ratio in the starter-1 phase was not affected by the different feeding strategies. In the starter-2 phase, the CON pullets had a higher water-to-feed ratio than the 20-ON and 30-TW pullets (*P* = 0.022), whereas the 20-TW pullets (1.78) did not differ from the pullets receiving the other treatments. The average water-to-feed ratio in the grower phase was 1.47, 1.54, 1.64, and 1.71 for the CON, 20-ON, 20-TW, and 30-TW pullets, respectively, showing a difference between all treatments (*P* < 0.001). The water-to-feed ratio in the developer phase was not affected by the different feeding strategies. The average water-to-feed ratio in the entire rearing period was 1.70, 1.68, 1.76, and 1.79 for the CON, 20-ON, 20-TW, and 30-TW pullets, respectively, showing a difference between all treatments (*P* = 0.003).

**Table 4:** The effects of different feeding strategies on the average water intake and the average water-to-feed ratio in the different phases and the entire rearing period.

Treatment^1^	Starter-1 (0–3 WOA^2^)		Starter-2 (3–6 WOA)		Grower (6–15 WOA)		Developer (15–20 WOA)		Rearing period (0-20 WOA)	
	Water intake (ml/b/d)	Water-to-feed ratio	Water intake (ml/b/d)	Water-to-feed ratio	Water intake (ml/b/d)	Water-to-feed ratio	Water intake (ml/b/d)	Water-to-feed ratio	Water intake (ml/b/d)	Water-to-feed ratio
CON	67.3	2.32	86.7^d^	1.89^a^	104.5^d^	1.47^d^	145.1^c^	1.62	106.4^d^	1.70^b^
20-ON	71.8	2.25	92.3^c^	1.69^b^	135.8^c^	1.54^c^	172.5^b^	1.59	128.8^c^	1.68^b^
20-TW	71.4	2.23	98.1^b^	1.78^ab^	143.7^b^	1.64^b^	182.6^b^	1.67	135.7^b^	1.76^a^
30-TW	70.2	2.18	103.8^a^	1.73^b^	160.2^a^	1.71^a^	201.0^a^	1.71	148.4^a^	1.79^a^
SEM	1.13	0.037	1.70	0.037	1.57	0.019	3.66	0.036	1.35	0.016
*P*-value	0.073	0.147	<0.001	0.022	<0.001	<0.001	<0.001	0.165	<0.001	0.003

a-d Means within a column with no common superscript differ (*P ≤* 0.05).

1 CON: control diet; 20-ON: 20% diluted diet fed once a day; 20-TW: 20% diluted diet fed twice a day; 30-TW: 30% diluted diet fed twice a day.

2 WOA: weeks of age.

### Total Daily Feed and Water Intake

**Figure 2:**
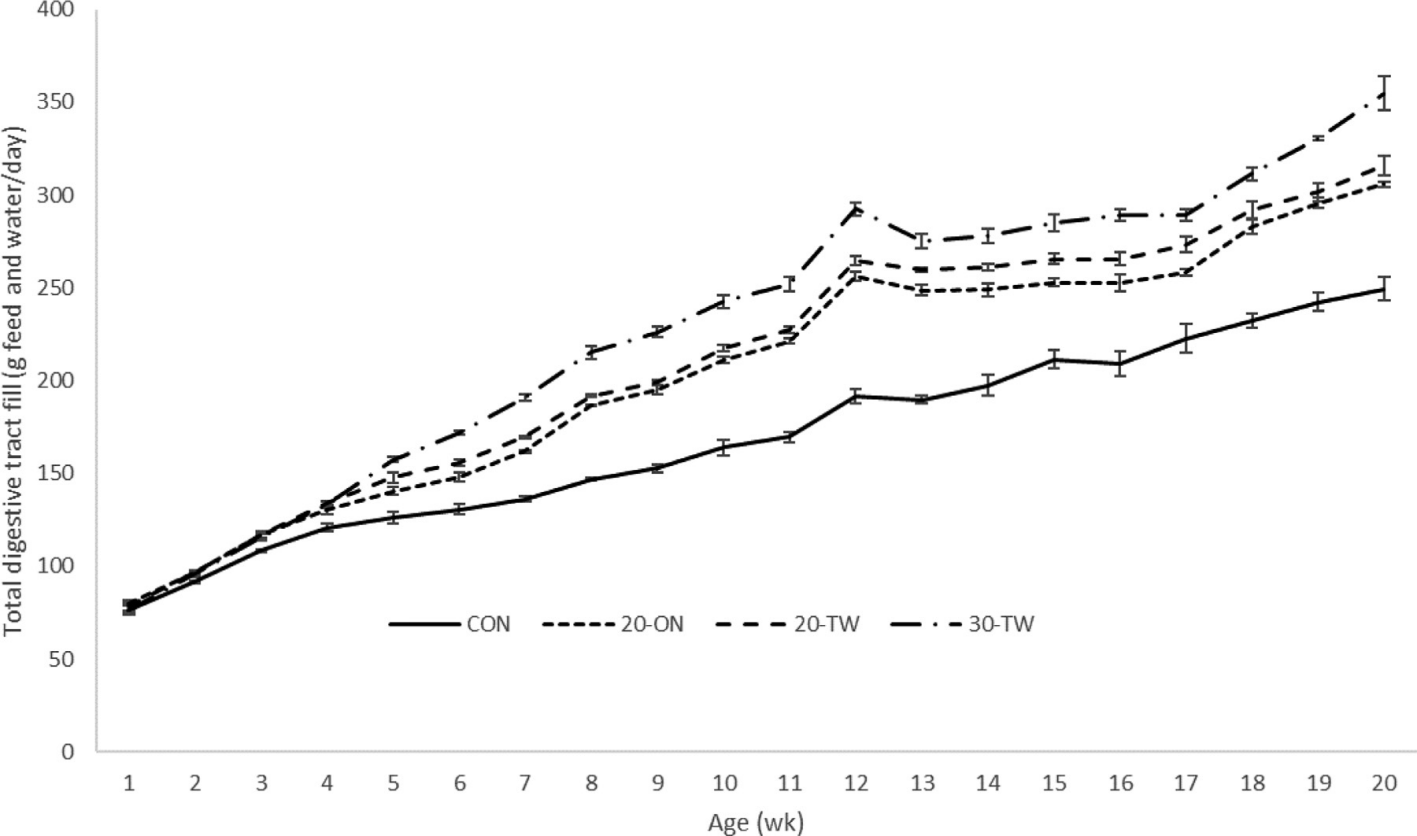
Effect of feeding strategies on the total daily feed and water intake (sum of total feed and water per day + SEM).

The total daily feed and water intake was the highest for the 30-TW pullets, followed by the 20-TW and 20-ON pullets, and was the lowest for the CON pullets (Figure 2). Over the entire rearing phase, the total daily feed and water intake was, compared with the CON pullets, 21.4%, 25.6%, and 36.0% higher for the 20-ON, 20-TW, and 30-TW pullets, respectively (*P* < 0.001).

### BW and BW Uniformity

No differences in average BW were found in the starter-1 phase (Table 5). During the starter-2 phase and grower phase, the 20-TW pullets had the lowest BW, whereas the 20-ON pullets had the highest BW in the developer phase. BW uniformity (CV%) was not affected by the different treatments in the starter-1 phase and starter-2 phase. In the grower phase, developer phase, and total rearing period, CV% was lower in the 20-TW and 30-TW pullets than in the pullets receiving the other treatments. In the grower phase, the 20-ON pullets had the highest CV% compared with all the other pullets, with no difference compared with the CON pullets in the developer phase. During the laying phase, no differences in BW and BW uniformity were found (data not shown).

**Table 5:** The effects of different feeding strategies on the average BW and the average BW uniformity (CV%) per phase and the entire rearing period.

Treatment^1^	Starter-1 (0–3 WOA^2^)		Starter-2 (3–6 WOA)		Grower (6–15 WOA)		Developer (15–20 WOA)		Rearing period (0–20 WOA)	
	BW (g)	CV%	BW (g)	CV%	BW (g)	CV%	BW (g)	CV%	BW (g)	CV%
CON	278	12.7	625^a^	12.0	1364^a^	12.2^b^	2190^b^	11.5^a^	1297^ab^	12.1^b^
20-ON	278	12.5	630^a^	12.9	1349^ab^	14.3^a^	2224^a^	12.7^a^	1299^a^	13.4^a^
20-TW	277	11.7	606^b^	10.7	1333^b^	11.1^c^	2181^b^	9.6^b^	1277^c^	10.7^c^
30-TW	282	12.0	630^a^	11.1	1355^ab^	11.2^c^	2164^b^	9.4^b^	1288^bc^	10.9^bc^
SEM	2.0	0.66	2.8	0.82	5.8	0.28	9.0	0.47	3.6	0.40
P-value	0.340	0.693	<0.001	0.292	0.025	<0.001	0.007	0.002	0.008	0.003

a-d Means within a column with no common superscript differ (*P ≤* 0.05).

1 CON: control diet; 20-ON: 20% diluted diet fed once a day; 20-TW : 20% diluted diet fed twice a day; 30-TW: 30% diluted diet fed twice a day.

2 WOA: weeks of age.

### Mortality Cause and Total Mortality

More pullets were graded from the 20-ON pens than from the 20-TW and 30-TW pens (*P* = 0.043), whereas the CON pullets did not differ from the other pullets (Table 6). More dead pullets were found in the CON pens than in the 20-ON and 20-TW pens (*P* = 0.028), whereas the 30-TW pullets did not differ from the pullets receiving the other treatments. A higher total mortality was found in the CON pullets than in the 20-ON, 20-TW and 30-TW pullets (*P* = 0.006).

**Table 6:** The effects of different feeding strategies on mortality cause, total mortality and grading at 20 WOA (% of the total number of placed pullets).

Treatment^1^	Legs	Culling	Pecking	Dead	Total mortality	Grading
CON	0.6	0.5	0.5	1.1^a^	2.7^a^	7.6^ab^
20-ON	0.5	0.4	0.0	0.5^b^	1.4^b^	11.3^a^
20-TW	0.6	0.2	0.0	0.2^b^	1.1^b^	6.2^b^
30-TW	0.4	0.2	0.0	0.6^ab^	1.2^b^	6.2^b^
SEM	0.16	0.17	0.25	0.17	0.26	1.19
*P*-value	0.643	0.687	0.436	0.028	0.006	0.043

a-b Means within a column with no common superscript differ (*P ≤* 0.05).

1 CON = control diet; 20-ON = 20% diluted diet fed once a day; 20-TW = 20% diluted diet fed twice a day; 30-TW = 30% diluted diet fed twice a day.

### Behavior

Approximately twice as many 20-TW and 30-TW pullets exhibited feeding behavior than CON pullets (*P* = 0.047), whereas the 20-ON pullets did not differ from the pullets receiving the other treatments (Table 7). There was no difference in percentage of pullets feeding at the different ages. The 20-TW and 30-TW pullets show more drinking than the CON and 20-ON pullets (*P* < 0.001). More pullets show drinking at 14 WOA than at 6 and 10 WOA (*P* = 0.010). Less 20-ON pullets showed foraging than the CON, 20-TW, and 30-TW pullets (8.4% vs. 12.7%, 11.9%, and 11.0%; *P* = 0.001). Number of pullets performing foraging increased with age from approximately 9% to 13% (*P* < 0.001). The different feeding strategies did not affect standing (*P* = 0.173) and comfort (*P* = 0.111). As they aged, more pullets showed standing and less walking (*P* < 0.001). The 20-TW and 30-TW pullets showed more sitting compared with the CON and 20-ON pullets (*P* < 0.001). With age, comfort behavior decreased from approximately 9% to 6% (*P* < 0.001). Feeding strategy and age did not affect dustbathing behavior (*P* > 0.10). Feather pecking was on average very low; however, this behavior was more observed in CON pullets, and more feather pecking was observed at 14 WOA. The CON and 20-ON pullets showed more object pecking (especially to the empty feeder) than the 20-TW pullets (*P* = 0.017). The 30-TW pullets did not differ from the pullets receiving the other treatments. More pullets pecking to the pecking blocks was found for the 20-TW pullets than for the CON pullets (*P* = 0.017), whereas the 20-ON and 30-TW pullets did not differ from the pullets receiving the other treatments. The CON pullets showed more aggressive pecking than the other pullets (*P* < 0.001).

**Table 7:** The effects of different feeding strategies on behavioral traits (% of time).^1^

Treatment^1^	Eat	Drink	Forag	Stand	Walk	Sit	Comf	Dustb	Feather	Object	Block	Aggressive
Feeding strategy												
CON^2^	12.6^b^	8.0^b^	12.7^a^	17.4	4.1	16.6^a^	7.7	1.0	0.1^a^	19.0^a^	0.3^b^	0.7^a^
20-ON^2^	21.7^ab^	7.9^b^	8.4^b^	13.7	3.4	17.9^a^	6.4	0.9	0.0^b^	18.8^a^	0.5^ab^	0.3^b^
20-TW^2^	23.2^a^	10.7^a^	11.9^a^	15.6	4.1	12.5^b^	6.9	0.9	0.1^ab^	13.1^b^	0.7^a^	0.4^b^
30-TW^2^	26.2^a^	11.2^a^	11.0^a^	13.3	4.1	11.5^b^	6.1	1.1	0.0^b^	14.1^ab^	0.5^ab^	0.4^b^
SEM	3.49	0.57	0.79	1.34	0.24	1.18	0.46	0.21	0.02	1.80	0.10	0.06
Age												
6 WOA	20.9	8.8^b^	9.0^b^	12.1^c^	6.9^a^	6.7^c^	9.2^a^	0.8	0.0^b^	25.0^a^	0.6	0.0^b^
10 WOA	21.5	8.3^b^	9.4^b^	5.8^d^	3.3^b^	28.1^a^	5.9^b^	1.2	0.0^b^	15.8^b^	0.6	0.0^b^
14 WOA	20.3	10.8^a^	13.2^a^	17.7^b^	2.9^bc^	13.7^b^	5.6^b^	1.0	0.1^a^	13.3^b^	0.5	0.9^a^
18 WOA	21.4	9.9^ab^	12.5^a^	24.5^a^	2.6^c^	9.9^c^	6.3^b^	0.8	0.0^b^	11.0^b^	0.3	1.0^a^
SEM	3.49	0.57	0.79	1.34	0.24	1.18	0.46	0.21	0.02	1.80	0.10	0.06
*P*-value												
Feeding strategy	0.047	<0.001	0.001	0.173	0.097	<0.001	0.111	0.936	0.002	0.017	0.017	<0.001
Age	0.990	0.010	<0.001	<0.001	<0.001	<0.001	<0.001	0.365	<0.001	<0.001	0.166	<0.001
Feeding × Age	0.986	0.844	0.704	0.988	0.938	0.701	0.935	0.900	0.982	0.987	0.714	0.866

a-c Means within a column and source without a common superscript differ significantly (*P ≤* 0.05).

1 Eat = pecking at the feed at the feeding pans; forag = foraging; drink = pecking at the nipple drinker; stand = standing; walk = walking; sit = sitting; comf = comfort behavior; dustb = dustbathing; feather = feather pecking; obj = object pecking; block = pecking at the peck blocks; aggressive = aggressive pecking.

2 CON = control diet; 20-ON = 20% diluted diet fed once a day; 20-TW = 20% diluted diet fed twice a day; 30-TW = 30% diluted diet fed twice a day. WOA = weeks of age.

**Figure 3:**
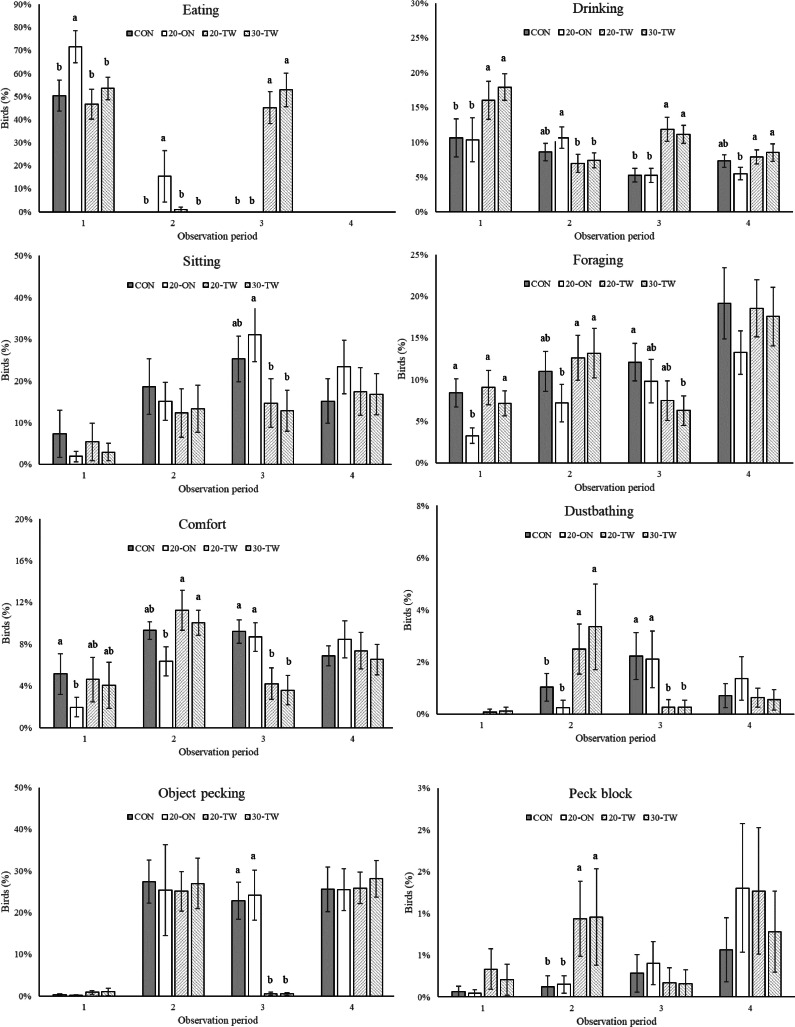
Effect of feeding strategies on the development of behavior over the 4 observation sessions. Means (± SEM) with no common superscript differ (*P* ≤ 0.05).

Significant interactions (*P* ≤ 0.05) between feeding strategy and observation period during the day were found for most types of behavior, and therefore figures of different types of behavior and observation periods are presented (Figure 3). The 20-ON pullets showed more eating behavior during the first and second observation periods than the other pullets. During the third observation period, the pullets fed twice a day (20-TW and 30-TW) showed more eating behavior than the pullets fed once a day (CON and 20-ON). The pullets fed twice a day (20-TW and 30-TW) showed more drinking behavior than the pullets fed once a day (CON and 20-ON) during the first and third observation periods. The 20-ON pullets showed more and less drinking behavior in the second and fourth periods, respectively, than the 20-TW and 30-TW pullets. The CON pullets showed no differences in drinking behavior in the second and fourth periods compared with the other pullets. During the third observation period, more sitting behavior was observed in the 20-ON pullets than in the 20-TW and 30-TW pullets, whereas the CON pullets showed no difference. No differences in sitting behavior were found between the first, second, and fourth observation periods. The 20-ON pullets showed less foraging behavior in the first and second observation periods than the pullets receiving the other treatments. More foraging behavior was observed in the third period in the CON pullets than in the 30-TW pullets, whereas the 20-ON and 20-TW pullets did not differ. The 20-ON pullets showed the least comfort behavior in the first and second observation periods. The CON pullets showed the most comfort behavior in the first observation period, whereas the 20-TW and 30-TW pullets showed the most comfort behavior in the second period. In the third period, the pullets fed once a day (CON and 20-TW) showed more comfort behavior than the pullets fed twice a day (20-TW and 30-TW). No differences were observed in the third period. Pullets fed twice a day (20-TW and 30-TW) showed more and less dustbathing behavior in the second and third observation periods, respectively, than the pullets fed once a day (CON and 20-ON). Less object pecking was observed in the third period for the pullets fed twice a day (20-TW and 30-TW) than the pullets fed once a day (CON and 20-ON). No differences in object pecking were observed in the first, second, and third observation periods. More pecking to the pecking blocks was observed in the second period in the pullets fed twice a day (20-TW and 30-TW) than in the pullets fed once a day (CON and 20-ON). No differences in pecking to the pecking blocks were found in the first, third, and fourth periods. No differences in any observation period were observed for standing, walking, feather pecking, and aggressive pecking behavior (graphs not shown).

### Novel Food Test

No differences in latency to approach the feeder pan were observed between the different feeding strategies. Fewer 20-ON, 20-TW, and 30-TW pullets than CON pullets approached the novel feeder pan after 1 min (*P* = 0.006) (Table 8). More CON pullets than 20-ON pullets approached the novel feeder pan after 2 min (*P* = 0.016), whereas the pullets fed twice a day (20-TW and 30-TW) did not differ from the pullets receiving the other treatments (*P* = 0.016). Feed intake was the highest (175.3 g/bird) for the CON pullets compared with the 20-ON, 20-TW, and 30-TW pullets, with 38.3, 67.0, and 89.8 g/bird, respectively (*P* = 0.002). Latency to approach the feeder pan was higher at 12 WOA than at 17 WOA (*P* = 0.019). Fewer pullets approached the feeder pan after 1 min and 2 min at 12 WOA than at 17 WOA (*P* < 0.001). Feed intake was lower at 12 WOA than at 17 WOA (*P* < 0.001). Latency to approach the feeder tended to be higher during the morning observation period than during the afternoon observation period (*P* = 0.062). No differences in the number of pullets that approached the feeder pan and in feed intake were found between the morning and afternoon observation periods.

**Table 8:** The effects of different feeding strategies on latency to approach the novel feeder (with novel feed) and on feed intake.

Treatment^1^	Latency to feeder (sec)	Nr of pullets after 1 min	Nr of pullets after 2 min	Feed intake (g/bird)
Feeding strategy				
CON	12.8	13.4^a^	17.9^a^	175.3^a^
20-ON	28.1	2.6^b^	5.8^b^	38.3^b^
20-TW	18.0	5.0^b^	12.5^ab^	67.0^b^
30-TW	27.6	7.8^b^	12.1^ab^	89.8^b^
SEM	6.81	1.84	2.25	20.73
Age				
12 WOA	30.7^a^	1.9^b^	5.2^b^	38.8^b^
17 WOA	12.6^b^	12.5^a^	18.9^a^	146.4^a^
SEM	4.81	1.30	1.59	14.62
Part of the day				
AM	27.4	4.5	8.7	37.5
PM	15.9	9.9	15.4	147.6
SEM	2.12	2.22	1.82	26.82
*P*-value				
Feeding strategy	0.338	0.006	0.016	0.002
Age	0.019	<0.001	<0.001	<0.001
Part of the day	0.062	0.229	0.119	0.100
Feeding × Age	0.956	0.066	0.335	0.354
Feeding × Part	0.769	0.203	0.507	0.041
Age × Part	0.403	0.392	0.285	0.111
Feeding × Age × Part	0.583	0.465	0.341	0.867

a-b Means within a column and source with no common superscript differ (*P ≤* 0.05).

1 CON = control diet; 20-ON = 20% diluted diet fed once a day; 20-TW = 20% diluted diet fed twice a day; 30-TW = 30% diluted diet fed twice a day. AM = before noon; PM = after noon. WOA = weeks of age.

### Production Performance

No differences in production performance were observed between the treatments (Table 9).

**Table 9:** The effects of different feeding strategies on production performance traits until 40 WOA.^1^

Treatment^2^	Total eggs (#)	Settable eggs (#)^3^	Small eggs (#)^4^	Double-yolk eggs (#)	Abnormal-shell eggs (#)^5^	Dirty eggs (#)	Floor eggs (%)	Egg weight (g)	Mortality (%)
CON	98.1	86.6	6.1	1.7	1.3	2.4	6.2	59.6	4.1
20-ON	97.7	86.2	5.9	1.7	1.4	2.7	3.6	59.8	5.0
20-TW	98.0	86.5	6.1	1.8	1.3	2.3	2.7	59.8	3.9
30-TW	99.6	87.9	6.5	1.7	1.2	2.4	2.8	59.7	3.2
SEM	1.01	1.17	0.23	0.10	0.08	0.22	0.01	0.13	0.80
*P*-value	0.566	0.734	0.391	0.769	0.728	0.724	0.128	0.839	0.503

1 Production performance traits calculated on a hen-housed basis (20 WOA).

2 CON = control diet; 20-ON = 20% diluted diet fed once a day; 20-TW = 20% diluted diet fed twice a day; 30-TW = 30% diluted diet fed twice a day.

3 Settable eggs = normal egg ≥ 50 gram.

4 Settable eggs = normal egg ˂ 50 gram.

5 Abnormal-shell eggs = misshaped, soft-shell, shell-less, and cracked eggs.

## DISCUSSION

The aim of this study was to evaluate the effects of different feeding strategies (diluted diets and feeding frequency) on the behavior and performance of broiler breeder pullets.

To reach the same BW at 20 WOA, the pullets fed the 20% and 30% diluted diets had a 22% and 30% higher feed allocation, respectively, than the pullets fed the control diets. Similar results were previously found in a study by Enting et al. (2007), who found a 12% and 25% higher feed intake (and similar energy intake) in pullets fed 12% and 23% diluted diets, respectively, than in pullets that were fed control diets. Moreover, feeding a 23%, 15%, and 16% diluted diet during the rearing phase resulted in a 24%, 14%, and 16.5% higher feed allocation, respectively, to meet the same BW at the end of the rearing phase in studies by Zuidhof et al. (2015), de los Mozos et al. (2017), and van Emous et al. (2021).

It was expected that total energy, CP, digestible lysine, and digestible methionine + cystine intake were equal for the different diluted diets to reach the same BW target at 20 WOA. The total energy and total digestible amino acids, however, were 2.0%, 1.6%, and 6.3% lower for the pullets fed the 20-ON, 20-TW, and 30-TW diets, respectively. This might be explained by the assumption of a zero energy and amino acid level of the wheat straw pellets during diet optimization, which is potentially an underestimation of the nutrient content of the diets. Additionally, dividing a single portion of standard feed into 2 portions during the day will improve the availability of nutrients, with potential positive effects on digestibility and utilization (Backhouse and Gous, 2006). Furthermore, feeding wheat straw pellets twice a day could have had an effect on gut integrity, as shown by Sweeney et al. (2022), who fed pullets every day instead of using a skip-a-day regime. Lastly, no information is available on the effect of the high fiber inclusion levels on digestibility. Sweeney et al. (2022) found increased villi height in the jejunum and ileum at 16 and 20 WOA, resulting in more efficient BW gain. Feeding pullets daily instead of using a skip-a-day regime divided nutrients over a longer period, but feeding twice a day instead of once a day during a shorter period might also improve the absorption of nutrients.

Due to the higher feed allocation, the average water intake of the pullets following the 20-ON, 20-TW, and 30-TW strategies was higher from week 3 onward. The average water-to-feed ratio in the starter-2 phase was lower for the 20-ON and 30-TW pullets than for the CON pullets. In contrast, in the grower phase, the water-to-feed ratio was the highest in the pullets following the 30-TW strategy and the lowest for the pullets fed the control diet, with the other treatments showing intermediate values. No effect was found in the developer phase. Over the entire rearing phase, the pullets fed twice a day (20-TW and 30-TW) had a higher water-to-feed ratio than the pullets fed once a day (CON and 20-ON) without any negative effects on litter quality, probably due to the high water binding capacity of the diluted diets. The higher water-to-feed ratio in the starter-2 phase was probably caused by the lower total amount of feed allowance, which was compensated by a relatively higher water intake to achieve gut fill. The higher water intake and water-to-feed ratio in the grower phase and entire rearing period might be explained by the higher sodium and chloride levels in the diet, as these nutrients were not diluted to support water intake and prevent constipation. Additionally, diluting diets with straw pellets results in higher dietary potassium levels. In combination with the higher feed allowance, this resulted in an average 38% and 57% higher daily potassium intake for the 20% and 30% diluted diets, respectively, in the entire rearing phase. The sodium and chloride levels in the diet were equal for all diets; however, the higher feed intake resulted in an average 22% and 30% higher daily sodium and chloride intake for the 20% and 30% diluted diets, respectively, in the entire rearing phase, resulting in an average 36% and 54% higher dEB for the 20% and 30% diluted diets, respectively, in the entire rearing phase. The higher dEB contributed to the higher water-to-feed ratio for the diluted diets.

Despite the pullets having unrestricted access to water during the entire light period, the average water-to-feed ratio was very low (1.65), especially compared with values found in previous studies (approximately 2.3) using unrestricted access to water (Nielsen et al., 2011; van Emous et al., 2021). An extremely high water-to-feed ratio (5.0) was found in an outdated study by Hocking et al. (1993). In that study, however, water was provided using an open water system and a high water level, which induces water intake and spillage. Despite double-checking the measuring equipment of the feed and water equipment, it is still unclear what the reason is for the low water-to-feed ratio in the current study.

The total daily feed and water intake was higher after 3 WOA for the pullets fed the diluted diets. It is hypothesized that due to the higher amount of feed and water in the digestive tract, the pullets fed the diluted diets experienced a higher satiety level and potentially had an increased gut passage time. The combination of these 2 factors has likely contributed to the observed improved welfare due to a lower feed intake motivation for the diluted groups, as shown in the novel feed test.

Except in the starter-1 phase, the average BW of the pullets differed between the different feeding strategies in the other phases (starter-2, grower, and developer) and the entire rearing period. Since the differences in SEMs were very small (on average 6.0) and inconsistent between phases in time, it is postulated that this might not represent a commercially relevant effect, or, if it did, the relevance in terms of the impact on the behavior and performance of the pullets was very small.

Average BW uniformity was identical for the different treatments during the starter-1 and starter-2 phases. This can be explained by the relatively short experimental period (6 wk), including 2 wk of feeding the same diet for all treatments. BW uniformity in the grower and developer phases and in the entire rearing period was the best (lowest CV%) for the pullets fed twice a day compared with the pullets fed once a day. This is in agreement with a study by van Emous et al. (2021), who observed an improved uniformity (11.5% vs. 14.2%) in pullets fed twice a day at 10 WOA; however, no difference was found at 20 WOA. The findings in the current study and the study by van Emous et al. (2021) differ, however, from those in a 20-y-old study by van der Haar and van Voorst (2001), who found a lower BW uniformity in pullets fed twice a day at 13 WOA. The discrepancy between the newer and older experiments may be explained by differences in feed structure (mash and pellets) and/or differences in breeds (Ross 308 and Ross 508). Van Emous et al. (2021) hypothesized that the improved BW uniformity was caused by physical differences in crop size and digestive tract length at a younger age. Heavier pullets at the beginning of the rearing phase and start of feed control (between 2 and 5 WOA), have relatively bigger crops compared with smaller pullets, which is a physical advantage for the amount of feed they can consume (Wehner and Harrold, 1982). When smaller pullets are fed twice a day, they have the possibility to consume continuously before the crop is filled to the maximum.

Total mortality (including grading) was the lowest in the pullets fed twice a day and the highest in the pullets fed the 20% diluted diets. This outcome is contrary to that of van der Haar and Voorst (2001) and van Emous et al. (2021), who found, respectively, a higher total mortality and no difference in total mortality. The lower total mortality in the current study for the pullets fed twice a day was caused by the lower percentage of graded pullets, caused by the better BW uniformity, and pullets dead in the pen. The reason for these differences is not clear, but it is speculated that this might be due to feeding twice a day. This feeding regime results in a more balanced distribution of nutrients throughout the day, as described by Backhouse and Gous (2006). Moreover, feeding twice a day is more in line with natural feeding and activity behavior (Collias and Collias, 1967), which may result in less stress, with positive effects on the immune system and health of the pullets.

Feeding twice a day resulted in more active pullets that showed more eating and drinking behavior and less sitting behavior. The outcome of the current study is contrary to the outcomes of previous studies by de Jong et al. (2005b) and Mens et al. (2022), who found no differences in eating and drinking behavior. Spending more time on eating is, however, in agreement with a study on breeders that were fed twice a day during the laying phase (van Emous and Mens, 2021). Feeding twice a day, however, affected eating patterns, as observed in the current study and previous studies on pullets (Mens et al., 2022) and breeders (van Emous and Mens, 2021; van Emous, 2023). In the current study, it was mentioned that pullets receiving only 1 meal per day did not react when the twice a day fed pullets in adjacent pens in the same room received the second meal. The pullets who received the second meal however showed similar active behavior with vocalizations, walking, running and flying as observed before the first meal. This observation was in line with a previous study with pullets fed once or twice a day (Mens et al., 2022). Despite these observations, it cannot be ruled out that the behavior of the animals was not mutually influenced by the different treatments.

Less stereotypic object pecking behavior was observed in the pullets fed twice a day compared with the pullets fed once a day. This is in close agreement with previous studies on pullets (de Jong et al., 2005b) and breeders (van Emous et al., 2021). De Jong et al. (2005b) observed a lower incidence of object pecking in pullets fed twice a day (on the litter) and concluded that this is an indication of improved welfare. This effect, however, was not found in pullets fed twice a day in a study by Mens et al. (2022).

Behavior patterns during the daylight period were different between the different treatments, and particularly pronounced differences were found between the pullets fed once and twice a day. Due to the second feeding moment at 1,100 h, the pullets fed twice a day were more active during the third observation period with eating and drinking. This resulted in less sitting, foraging, comfort, and dustbathing behavior during the third observation period. This is in agreement with a study by Mens et al. (2022), who also observed that pullets showed less sitting, foraging, and comfort behavior after the second feeding moment. Because of the different availability of feed, the pullets fed twice a day changed their behavior during the day. More foraging, comfort, and dustbathing behavior was observed in the second observation period. There was no difference in eating behavior in the first observation period, because the pullets fed twice a day received less feed at that time than the control pullets and the pullets fed 20% diluted diets. Object pecking was the lowest in the pullets fed twice a day during the third observation period directly after the second feeding moment. This is contrary to the findings of Mens et al. (2022), who did not find differences in object pecking in pullets after the second feeding moment. Our findings on object pecking after the second feeding moment are, however, in line with previous findings of studies on breeders fed twice a day (van Emous and Mens, 2021; van Emous, 2023).

Latency to approach the novel feeder (with novel feed) is an indicator of the motivation of the pullets to explore a novel object as they must overcome their fear (Nielsen et al., 2011). These authors suggested that pullets that are hungrier show a shorter latency to approach the feeder, with more pullets approaching the feeder after a certain time period. There was no difference in the latency to approach the novel feeder; however, differences were observed in the number of pullets approaching the novel feeder and the feed intake from the novel feeder. After 1 min, fewer pullets fed the diluted diets (once or twice a day) approached the novel feeder (and novel feed) than the pullets fed the standard diet once a day. After 2 min, however, this difference disappeared. In the study by Mens et al. (2022), fewer pullets were observed at the novel feeder after 2 and 2.5 min; however, no differences were found after 0.5, 1, 1.5, and 3 min. In the current study, more pullets approached the novel feeder at 17 WOA than at 12 WOA, which can be an indication of a higher motivation to approach a novel object due to the higher feed control.

The pullets in the pens with adjusted feeding strategies showed a lower feed intake than the pullets on the control diets, which is in disagreement with the study by Mens et al. (2022), who did not find differences in feed intake between pullets fed once and twice a day. Since a higher feed intake during the novel food test is associated with a higher level of hunger (Nielsen et al., 2011), the lower intake implicates increased welfare and a lower “hunger feeling” for the pullets with the diluted feeding strategies.

## CONCLUSIONS

In conclusion, feeding breeders with adjusted feeding strategies (feeding twice a day and feeding up to 30% diluted diets) resulted in improved behavior and welfare. This was expressed in decreased stereotypic pecking behavior and lower feed intake motivation with the novel feed test, with no negative effects on production performance. In addition, feeding pullets twice a day improved BW uniformity and decreased mortality. Future research should focus on the effect of a higher level of dilution and more feeding moments a day, in combination with water-holding capacity and gut fill, on performance and welfare.

## DISCLOSURES

The authors declare that they have no known competing financial interests or personal relationships that could have appeared to influence the work reported in this paper.
